# RESPONDING TO THE DIVERSE NEEDS OF STUDENT CAREGIVERS: RECOMMENDATIONS FOR CAMPUSES AND COMMUNITY PARTNERSHIPS

**DOI:** 10.1093/geroni/igae098.1571

**Published:** 2024-12-31

**Authors:** Katherina Nikzad-Terhune, Allyson Graf, Lydia Manning

**Affiliations:** Northern Kentucky University, Lexington, Kentucky, United States; Northern Kentucky University, Highland Heights, Kentucky, United States; Dele Health Tech, Downers Grove, Illinois, United States

## Abstract

Informal caregiving continues to steadily increase, warranting attention to the impact of being a caregiver. A growing demographic of caregivers that have received less attention are student caregivers. Currently, five million adult students attending post-secondary education are also caregivers of adults, resulting in negative impacts on academic success, mental health, and retention. Furthermore, the characteristics and experiences of student caregivers are diverse, thus prompting a need to better understand their experiences. Rooted in the age-inclusivity in higher education model, this presentation examines mixed methods data from a campus-wide study that assessed the prevalence and impact of unpaid caregiving on students. Survey results indicated that 16.9% (n = 241) of student respondents identified as a caregiver. Scores on the Adult Carers’ Quality of Life scale (Elwick et al., 2010) revealed that lack of support (46-48.8%) and financial matters surrounding caregiving (25.7-27.2%) place student caregivers at risk for low quality of life. Qualitative data from focus groups (N = 12) provided further explanation and clarification of the data. We found that student caregivers encompass a range of challenges, emotions, and coping strategies. The experiences of student caregivers highlight the need for greater recognition, understanding, and support within educational institutions. Based on the study findings, specific recommendations will be made for universities to better recognize the diverse needs of their student caregivers, and how to respond with support, resources, and empowerment. Additionally, recommendations for partnering with community agencies in the pursuit of supporting student caregivers will be highlighted.

